# Metabolic Syndrome and Its Components Are Associated with New-Onset Hyperuricemia in a Large Taiwanese Population Follow-Up Study

**DOI:** 10.3390/nu15051083

**Published:** 2023-02-21

**Authors:** Yen-Chieh Tu, Yi-Hsueh Liu, Szu-Chia Chen, Ho-Ming Su

**Affiliations:** 1 Graduate Institute of Clinical Medicine, College of Medicine, Kaohsiung Medical University, Kaohsiung 807, Taiwan; 2 Division of Cardiology, Department of Internal Medicine, Kaohsiung Medical University Hospital, Kaohsiung Medical University, Kaohsiung 807, Taiwan; 3 Department of Internal Medicine, Kaohsiung Municipal Siaogang Hospital, Kaohsiung Medical University Hospital, Kaohsiung Medical University, Kaohsiung 812, Taiwan; 4 Division of Nephrology, Department of Internal Medicine, Kaohsiung Medical University Hospital, Kaohsiung Medical University, Kaohsiung 807, Taiwan; 5 Faculty of Medicine, College of Medicine, Kaohsiung Medical University, Kaohsiung 807, Taiwan; 6 Research Center for Precision Environmental Medicine, Kaohsiung Medical University, Kaohsiung 807, Taiwan

**Keywords:** follow-up, Taiwan Biobank, hyperuricemia, metabolic syndrome, metabolic syndrome components

## Abstract

The prevalence rate of hyperuricemia remains high in Taiwan, at 21.6% in men and 9.57% in women. Both metabolic syndrome (MetS) and hyperuricemia can cause many complications; however, few studies have evaluated the correlation between MetS and hyperuricemia. Therefore, in this observational cohort study, we explored associations between metabolic syndrome (MetS) and its components and new-onset hyperuricemia. Of 27,033 individuals in the Taiwan Biobank who had complete follow-up data, we excluded those with hyperuricemia at baseline (*n* = 4871), those with gout at baseline (*n* = 1043), those with no data on baseline uric acid (*n* = 18), and those with no data on follow-up uric acid (*n* = 71). The remaining 21,030 participants (mean age 50.8 ± 10.3 years) were enrolled. We found a significant association between new-onset hyperuricemia with MetS and the components of MetS (hypertriglyceridemia, abdominal obesity, low high-density lipoprotein cholesterol, hyperglycemia, and high blood pressure). Furthermore, compared to those without any MetS components, those with one MetS component (OR = 1.816), two MetS components (OR = 2.727), three MetS components (OR = 3.208), four MetS components (OR = 4.256), and five MetS components (OR = 5.282) were significantly associated with new-onset hyperuricemia (all *p* < 0.001). MetS and its five components were associated with new-onset hyperuricemia in the enrolled participants. Further, an increase in the number of MetS components was associated with an increase in the incidence rate of new-onset hyperuricemia.

## 1. Introduction

The prevalence of hyperuricemia not only differs geographically across the world but also according to the economic development of the country. It has been reported that around 20% of men and women have hyperuricemia in the US [1] as well as up to 30% of the elderly population in Taiwan [2]. Purine metabolism results in the production of urate, which is mainly synthesized in the liver [3]. Urate metabolism in tissues is usually negligible under normal physiological conditions, and most excretion takes place in the gut and kidneys. Hyperuricemia is defined as the overproduction of uric acid, and it can be the result of a diet rich in purine and an increase in the degradation and metabolism of purine. Other possible causes of hyperuricemia include excessive alcohol consumption, acute chronic kidney disease leading to a reduction in uric acid excretion, the use of diuretics, hyperparathyroidism, acidosis, hypothyroidism, and lead poisoning. Factors resulting in increased urate synthesis (glycogen storage diseases, excessive ethanol/seafood consumption) or the decreased clearance of urate (sarcoidosis, use of diuretics) contribute to the development of hyperuricemia [4]. Hyperuricemia has been associated with gout, chronic kidney disease, hypertension, atrial fibrillation [5], myocardial infarction, and stroke. Therefore, studies analyzing the factors potentially causing a high serum uric acid level are warranted.

Treatment with xanthine oxidase inhibitors has been shown to be a safe and effective strategy to lower levels of uric acid and manage chronic hyperuricemia; however, pharmacogenetics have been shown to strongly modify the efficacy of uricosuric agents [6]. Certain factors, including increased body mass index (BMI), hyperglycemia, high blood pressure, and kidney disease, have been associated with the development of hyperuricemia [7,8]. A previous study evaluated short-term interactions between uric acid, low-density lipoprotein (LDL) cholesterol, and incident hypertension, and found that the presence of suboptimal uric acid and LDL cholesterol levels were associated with an elevated risk of developing hypertension [9]. In addition, Cicero et al. investigated the association between uric acid and the prevalence and 4-year incidence of metabolic syndrome (MetS) in older, overall healthy subjects, and it was found that hyperuricemia appeared to be a highly prevalent component of MetS, especially in those with the most severe forms, as well as a risk factor for developing MetS [10].

The importance of MetS and its complications has also been increasingly recognized. The prevalence of MetS is similar for men (24.0%) and women (23.4%) in the United States [11], compared to a lower rate of 15.7% in Taiwan [12]. MetS has been shown to increase the risk of developing type 2 diabetes mellitus (DM) [13], cardiovascular disease [14], chronic kidney disease [15], polycystic ovary syndrome [16], and obstructive sleep apnea [17], and a positive correlation between MetS and hyperuricemia has also been shown in previous studies [18,19]. Increased oxidative stress resulting from hyperuricemia in adipocytes may potentially play a role in the development of MetS [20]. However, large-scale studies investigating the association between MetS and hyperuricemia are lacking. To address this research gap, we conducted this longitudinal study with a large cohort of Taiwanese adults to explore correlations between MetS and its components and new-onset hyperuricemia.

## 2. Materials and Methods

### 2.1. Ethical Declaration

This study was conducted following the Declaration of Helsinki and was approved by the Institutional Review Board (IRB) of Kaohsiung Medical University Hospital (KMUHIRB-E(I)-20210058). Ethical approval for the Taiwan Biobank (TWB) was granted by the IRB on Biomedical Science Research, Academia Sinica, Taiwan, and the TWB Ethics and Governance Council.

### 2.2. TWB

The Taiwan government established the TWB to collect data on citizens aged 30–70 years who were enrolled around Taiwan for biomedical and epidemiological research purposes. Extensive genome and phenotype data were obtained during enrolment and follow-up. Fasting blood and urine samples were obtained to measure glucose, uric acid, hemoglobin, triglycerides, total/LDL, and high-density lipoprotein [HDL] cholesterol. In addition, physical examinations were performed to record data on body height/weight [BH/BW], waist circumference [WC], and hip circumference [HC]. Moreover, structured questionnaires were used to obtain information on histories of diabetes mellitus [DM], gout, hypertension, and smoking/alcohol habits, along with sex and age [21,22].

BMI was calculated as BW/BH^2^, and the 4-variable MDRD formula was used to calculate the estimated glomerular filtration rate (eGFR) [23]. Blood pressure (BP) was measured by researchers with an electronic device after the participants had abstained from exercising, consuming caffeine-related items, and smoking for a minimum of 30 min. Three BP measurements were recorded for each participant, separated by a 1–2 min break, and the average value was included in the analysis. The “Physical Fitness 333 Plan” criteria were used to define regular exercise as promoted by the Ministry of Education in Taiwan, which was defined as at least 30 min of exercise three times a week [24].

### 2.3. Definition of New-Onset Hyperuricemia

The participants were defined as having new-onset hyperuricemia if they were found to have an elevated serum uric acid level (>7.0 mg/dL in men; >6.0 mg/dL in women) during follow-up.

### 2.4. Definition of MetS

The NCEP-ATP III definition of MetS was adopted [25] with Asian-modified criteria [26] and required the presence of at least three of the following: (1) hyperglycemia, defined as fasting whole-blood glucose ≥ 110 mg/dL or a diagnosis of DM; (2) triglycerides ≥ 150 mg/dL; (3) HDL cholesterol < 50 mg/dL for women and <40 mg/dL for men; (4) systolic/diastolic BP ≥ 130/85 mmHg, hypertension diagnosis, or prescription for anti-hypertensive drugs; (5) abdominal obesity, defined as a WC > 80/90 cm in women/men.

### 2.5. Study Participants

**Figure 1:**
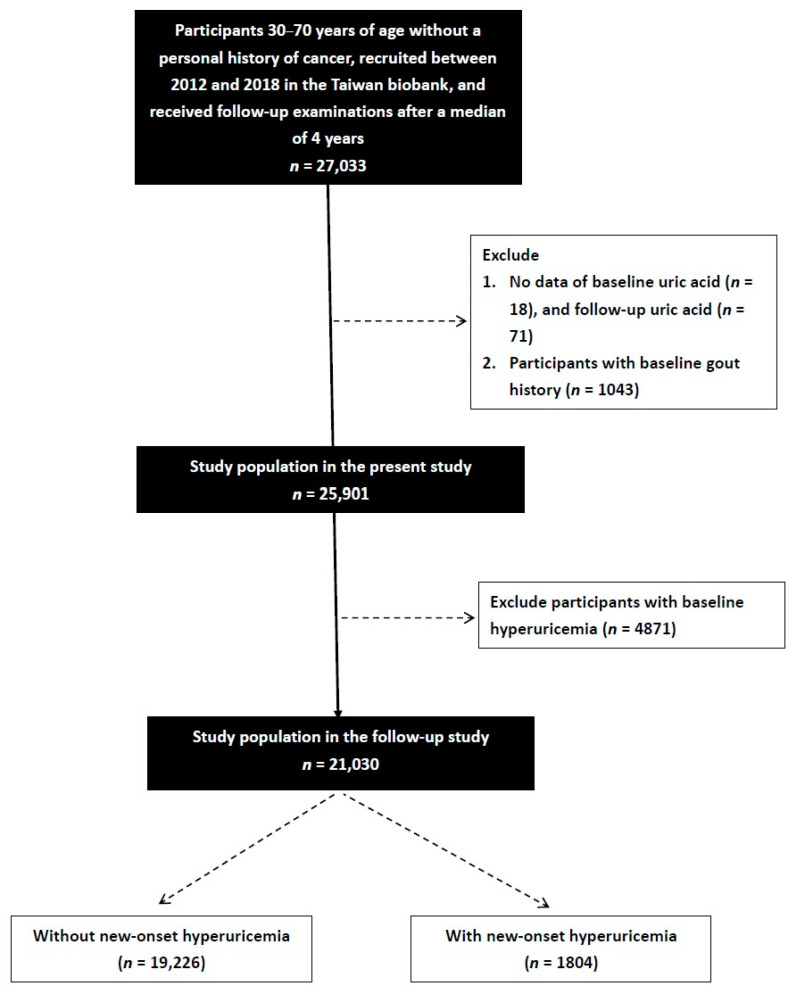
Flowchart of the study population.

Of 27,033 enrollees (males: 9555; females: 17,478) identified in the TWB (median follow-up period, 4 years), those with baseline hyperuricemia (*n* = 4871) or gout (*n* = 1043), and those who did not have data on baseline (*n* = 18) or follow-up uric acid (*n* = 71), were excluded. The remaining 21,030 participants provided written informed consent and were included in the study (Figure 1).

### 2.6. Study Design

This study was an observational cohort study.

### 2.7. Statistical Analysis

Categorical variables are expressed as numbers and percentages, and differences between them were analyzed using chi-square tests. Continuous variables are expressed as mean ± standard deviation, and differences between them were analyzed using independent t-tests. We have further performed multicollinearity analyses. The variance inflation factor (VIF) was used to detect multicollinearity in the regression model. Explanatory variables having a VIF of ≥5 indicated a multicollinearity problem. In our model, it was shown that the VIF of each variable was <5. Associations between MetS and its components and new-onset hyperuricemia were analyzed using multivariable logistic regression analyses and are presented as odds ratios (ORs) with 95% confidence intervals (CIs). Comparisons among groups according to the number of MetS components were made using a one-way analysis of variance. A *p*-value < 0.05 was considered statistically significant. All statistical analyses were conducted using SPSS version 19.0 for Windows (IBM Corp., Armonk, NY, USA).

## 3. Results

The mean age of the 21,030 enrolled participants was 50.8 ± 10.3 years, and 6286 were male. At follow-up, 1804 (8.6%) participants had developed new-onset hyperuricemia and 19,226 (91.4%) did not.

### 3.1. Comparison of the Participants Who Did and Did Not Develop New-Onset Hyperuricemia

Compared to the participants who did not develop new-onset hyperuricemia, those who did develop new-onset hyperuricemia had higher rates of smoking and alcohol intake, hypertension, and DM; were older; were predominantly male; had higher systolic/diastolic BP, HC, BW, BH, WC, BMI, uric acid, fasting glucose, hemoglobin, triglycerides, total cholesterol, and LDL cholesterol; and lower HDL cholesterol and eGFR (Table 1). In addition, the new-onset hyperuricemia group had a higher prevalence of MetS and its components (high BP, low HDL cholesterol, hypertriglyceridemia, abdominal obesity, and hyperglycemia).

### 3.2. Association of MetS and New-Onset Hyperuricemia

Multivariable logistic regression analysis showed that, after adjusting for age, sex, smoking, alcohol consumption, uric acid, hemoglobin, total cholesterol, LDL cholesterol, and eGFR, there were significant associations among old age (*p* = 0.003), sex (*p* < 0.001), alcohol consumption (*p* < 0.001), high uric acid (*p* < 0.001), low total cholesterol (*p* = 0.015), high LDL-cholesterol (*p* = 0.021), low eGFR (*p* = 0.003), and MetS (OR = 1.493; 95% CI = 1.312–1.700; *p* < 0.001) and new-onset hyperuricemia (Table 2).

### 3.3. Associations among MetS Components with New-Onset Hyperuricemia

**Figure 2:**
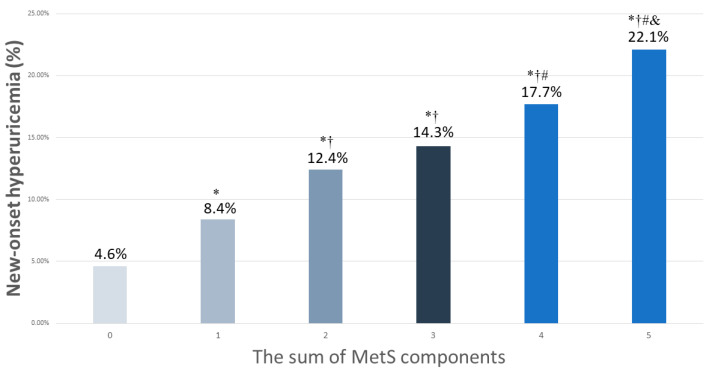
The percentage of new-onset hyperuricemia among 6 study groups, according to the sum of MetS components (0–5). * *p* < 0.05 compared to MetS number 0; † *p* < 0.05 compared to MetS number 1; # *p* < 0.05 compared to MetS number 2; & *p* < 0.05 compared to MetS number 3.

The participants were classified according to the number of MetS components (0 to 5) into six groups. There were 8069, 6432, 3902, 1827, 660, and 140 participants in the six groups, respectively. The rates of new-onset hyperuricemia in these six groups were 4.6%, 8.4%, 12.4%, 14.%, 17.7%, and 22.1%, respectively (Figure 2). The highest prevalence of new-onset hyperuricemia was found among the participants with five MetS components. Compared to the participants with no components, those with 1–5 components had higher rates of new-onset hyperuricemia (all *p* < 0.001). Compared to the participants with 1 component, those with 2–5 components had higher new-onset hyperuricemia rates (all *p* < 0.001). Compared to the participants with 2 components, those with 4–5 components had higher new-onset hyperuricemia rates (*p* < 0.001, *p* = 0.001, respectively). Further, the participants with 5 components had a higher new-onset hyperuricemia rate than those with 3 components (*p* = 0.020).

Associations among the number of MetS components and new-onset hyperuricemia using multivariable logistic regression analysis are shown in Table 3. Compared to the participants with no components, those with 1 component (OR = 1.413; 95% CI = 1.222–1.634; *p* < 0.001), 2 components (OR = 1.918; 95% CI = 1.647–2.233; *p* < 0.001), 3 components (OR = 1.915; 95% CI = 1.597–2.296; *p* < 0.001), 4 components (OR = 2.428; 95% CI = 1.910–3.100; *p* < 0.001), and 5 components (OR = 3.593; 95% CI = 2.281–5.658; *p* < 0.001) were significantly associated with new-onset hyperuricemia.

### 3.4. Associations among the MetS Components with New-Onset Hyperuricemia

Multivariable analysis showed that the participants with abdominal obesity (presented as ORs and 95% CIs) (1.180; 1.134–1.229), hypertriglyceridemia (1.293; 1.233–1.355), low HDL cholesterol (1.185; 1.135–1.236), hyperglycemia (1.136; 1.075–1.201), and high BP (1.167; 1.118–1.217) were significantly associated with new-onset hyperuricemia (all *p*-values < 0.001; Table 4).

## 4. Discussion

In this follow-up study of a large Taiwanese cohort, we found that MetS and its five components were associated with new-onset hyperuricemia. Furthermore, we found that the incidence rate of new-onset hyperuricemia increased as the number of MetS components increased.

A positive correlation between MetS and hyperuricemia has been reported in previous studies [18,19]. One longitudinal cohort study with 3247 participants found that MetS and its components could increase the risk of hyperuricemia in Chinese adults aged 60 years or older [27]. In that study, hypertension was the most important risk factor, and subjects with hypertension in combination with DM and high triglycerides had the highest risk of developing hyperuricemia [27]. A four-year follow-up cohort study using cross-lagged panel analysis found a bidirectional relationship between MetS and new-onset hyperuricemia [28]. Moreover, two components of MetS, systolic BP, and triglycerides, were also found to share this bidirectional relationship with hyperuricemia [28]. The authors suggested that the excessive fat storage in MetS upregulated the activity of xanthine oxidoreductase, which in turn increased the secretion of uric acid. This phenomenon has been reported to be most pronounced in individuals with obesity, low HDL cholesterol, hyperglycemia, or elevated triglycerides [29].

We also found that abdominal obesity was associated with new-onset hyperuricemia. A causal relationship was found between BMI and the risk of hyperuricemia in a large cohort study using mendelian randomization analysis, in which the risk of hyperuricemia increased by 7.5% (3.9% to 11.1%) with one standard deviation increase in BMI [30]. One study in China also found a positive correlation between newly diagnosed hyperuricemia and abdominal obesity regardless of sex [8]. However, the prevalence of hyperuricemia has been reported to be higher in obese men than women, possibly due to the effect of estrogen and progesterone on increasing urate excretion and reducing reabsorption [31]. In addition, abdominal obesity (defined as WC ≥ 85.0 cm for males and ≥ 80.0 cm for females) showed an OR of 2.26 (1.88, 2.73) for men and 1.96 (1.61, 2.39) for women to develop new-onset hyperuricemia [31]. The overproduction and impaired excretion of uric acid may serve as the link between obesity and hyperuricemia, and it has been hypothesized that the location of fat accumulation may also affect the development of hyperuricemia [32]. Increased leptin secretion, which is one of the cytokines produced by adipose tissue, may also result in hyperuricemia by decreasing renal uric acid excretion [33].

Another important finding of this study is the association between hypertriglyceridemia and new-onset hyperuricemia. A prospective study with 6 years of follow-up data also found that hypertriglyceridemia was a strong and independent risk factor for developing hyperuricemia [34]. Moreover, the results did not differ regardless of whether the triglyceride level at 6 years or the change in triglyceride level was used for analysis. Various previous studies also support the significant correlation between hypertriglyceridemia and hyperuricemia [35]. However, the mechanisms underlying the association between hypertriglyceridemia and hyperuricemia are not fully understood. Decreased glyceraldehyde 3-phosphate dehydrogenase activity has been observed in individuals with hyperlipidemia, resulting in enhanced uric acid synthesis [36]. Triglycerides can also cause stenosis of small renal vessels under long-term conditions of dyslipidemia, ultimately impairing the ability to excrete urate [37].

The fourth important finding of this study is the association between low HDL cholesterol and new-onset hyperuricemia. A prospective study of 1508 participants found that rather than decreasing the level of HDL, hyperuricemia impaired the antioxidative/ anti-inflammatory effect of HDL itself [38]. While hyperuricemia and low serum HDL both have a proinflammatory effect and contribute to the formation of atherosclerosis, the exact mechanism underlying their association remains to be elucidated. Previous studies have shown an inverse association between serum uric acid concentration and HDL cholesterol level [39]. In one study, hyperuricemia was associated with not only low HDL cholesterol but also the presence of denser, smaller particles of HDL [40]. While uric acid has an antioxidant effect, smaller HDL particles have been closely linked to high oxidative stress and reduced paraoxonase activity, reflecting a decreased antioxidative effect [41]. It has, therefore, been hypothesized that hyperuricemia may act as a compensatory response to the elevated oxidative stress resulting from a low HDL cholesterol level and smaller HDL particle size [40].

We also found that hyperglycemia was associated with new-onset hyperuricemia. This is consistent with the findings of Yoo et al., who found that hyperglycemia was a risk factor for the development of hyperuricemia [42]. Hyperuricemia has been shown to cause insulin resistance and the dysregulation of glucose metabolism through β-cell injury and dysfunction [15]. However, conflicting data have been reported regarding the association between hyperglycemia and hyperuricemia. While it has been observed that hyperglycemia remains a risk factor for the development of hyperuricemia [42], a study with 2374 participants in China did not find a statistically significant correlation between the two [43], suggesting future studies are needed to further clarify their interaction. In that study, among the group with a normal serum glucose level, the uric acid level increased as the fasting glucose concentration increased. Andrade et al. reported an increase in the prevalence of hyperuricemia from controls (3.9%) to those with euglycemic hypertension (7.6%) to those with prediabetes (14.0%), with a significant difference between the patients with prediabetes and the controls. In addition, the prevalence of hyperuricemia in diabetic patients was 11.4%, which was also significantly different compared to the control group [44]. The mechanism underlying the association between hyperglycemia and hyperuricemia may be related to proximal tubules. Nephron hypertrophy occurs during the early stage of dysglycemia, possibly to prevent the loss of glucose [45]. However, increased proximal glucose reabsorption may affect the level of serum glucose and worsen the retention of urate. In addition, a high level of insulin is common in patients with insulin resistance, and this may play a role in sodium and urate retention at more distant sites along nephrons [46].

Finally, we found that high BP was associated with new-onset hyperuricemia. It has become increasingly recognized that hypertension is strongly correlated with hyperuricemia; however, the direction of the causal effect still remains under debate [4,47]. There is increasing recognition that hyperuricemia is strongly correlated with hypertension. Hyperuricemia has not only been observed to be associated with the risk of hypertension and metabolic syndrome but also increased carotid intima-media thickness [48]. The PAMELA study, a large epidemiological study conducted in Italy, found that the risks of home and ambulatory hypertension increased by 34% and 29%, respectively, for each 1 mg/dL increase in serum uric acid concentration [49]. Animal studies have shed light on the possible mechanism behind the correlation between hyperuricemia and hypertension. In the acute phase, hyperuricemia impairs the release of endothelium-derived nitric oxide [50] and increases oxidative stress in macula densa cells, which in turn leads to renal vaso-constriction and ischemia [51]. Prolonged changes in inflammatory microvascular results in glomerular afferent arteriolopathy and interstitial inflammation, further leading to the development of hypertension [52]. Using cross-lagged path analysis, Han et al. reported that insulin resistance resulting from hyperuricemia may play a role in the development of hypertension [47]. A bidirectional relationship between systolic BP and serum uric acid was found in a longitudinal study in China [28]. One large, population-based cohort study of 15,792 individuals in the US identified several risk factors for developing hyperuricemia, of which, hypertension carried a 1.65-fold increased risk [53]. Hypertension results in elevated systemic and renal vascular resistance, which in turn decreases renal blood flow and subsequently increases urate reabsorption [27,35,54]. Renal microvascular injury resulting from hypertension has also been reported to lead to impaired urate excretion and increased urate synthesis [54].

This study is strengthened by the large-scale investigation and follow-up. Nevertheless, there are also some limitations. First, information on drugs that may influence the presence of hyperuricemia is lacking in the TWB. Consequently, the association between MetS and hyperuricemia may have been underestimated. Second, all enrollees were ethnically Chinese, and hence, our findings may not be generalizable to other groups. In addition, because it is an observational cohort study, this study cannot determine a causal relationship between MetS and hyperuricemia. Finally, not all TWB enrollees returned for follow-up assessments, and this may have led to sample bias.

In conclusion, new-onset hyperuricemia was associated with MetS and its five components in a large Taiwanese population. Further, the incidence of new-onset hyperuricemia increased with the increase in the number of MetS components.

## Figures and Tables

**Table 1:** Comparison of clinical characteristics among participants according to new-onset hyperuricemia in study participants.

Characteristics	New-Onset Hyperuricemia (-) (*n* = 19,226)	New-Onset Hyperuricemia (+) (*n* = 1804)	*p*
Age (year)	50.7 ± 10.3	52.6 ± 10.1	<0.001
Male (%)	29.0	39.7	<0.001
DM (%)	4.4	7.3	<0.001
Hypertension (%)	9.6	17.5	<0.001
Smoking history (%)	21.6	28.5	<0.001
Alcohol history (%)	2.0	4.9	<0.001
Regular exercise habits (%)	48.4	48.7	0.763
Systolic BP (mmHg)	115.4 ± 17.3	121.7 ± 17.9	<0.001
Diastolic BP (mmHg)	71.0 ± 10.6	74.7 ± 10.7	<0.001
Body height (cm)	160.5 ± 7.8	161.2 ± 8.2	<0.001
Body weight (kg)	60.3 ± 10.6	65.5 ± 11.1	<0.001
Waist circumference (cm)	81.2 ± 9.1	85.8 ± 9.0	<0.001
Hip circumference (cm)	94.7 ± 6.3	97.1 ± 6.7	<0.001
BMI (kg/m^2^)	23.3 ± 3.2	25.1 ± 3.4	
Laboratory parameters			
Uric acid (mg/dL)	4.9 ± 1.0	5.8 ± 0.8	<0.001
Fasting glucose (mg/dL)	95.0 ± 19.7	99.3 ± 25.2	<0.001
Hemoglobin (g/dL)	13.5 ± 1.5	13.9 ± 1.5	<0.001
Triglyceride (mg/dL)	101.2 ± 72.2	129.4 ± 81.0	<0.001
Total cholesterol (mg/dL)	193.8 ± 34.7	196.6 ± 35.4	0.001
HDL cholesterol (mg/dL)	56.3 ± 13.3	51.5 ± 12.3	<0.001
LDL cholesterol (mg/dL)	119.8 ± 31.0	124.2 ± 31.2	<0.001
eGFR (mL/min/1.73 m^2^)	113.5 ± 24.7	105.0 ± 24.0	<0.001
MetS (%)	11.5	22.7	<0.001
MetS numbers	1.05 ± 1.12	1.62 ± 1.24	<0.001
MetS component			
Abdominal obesity (%)	37.4	52.4	<0.001
Hypertriglyceridemia (%)	14.0	27.1	<0.001
Low HDL cholesterol (%)	22.6	33.1	<0.001
Hyperglycemia (%)	8.7	13.0	<0.001
High blood pressure (%)	22.0	36.0	<0.001

Abbreviations: DM, diabetes mellitus; BP, blood pressure; BMI, body mass index; HDL, high-density lipoprotein; LDL, low-density lipoprotein; eGFR, estimated glomerular filtration rate; MetS, metabolic syndrome.

**Table 2:** Association of MetS and new-onset hyperuricemia using logistic regression analysis.

Variables	Age and Sex Adjusted		Multivariable *	
	Odds Ratio (95% CI)	*p*	Odds Ratio (95% CI)	*p*
Age (per 1 year)	1.013 (1.008–1.018)	< 0.001	1.008 (1.003–1.014)	0.003
Female (vs. male)	0.643 (0.582–0.711)	< 0.001	4.697 (3.900–5.657)	<0.001
Smoking history	-	-	1.047 (0.909–1.205)	0.525
Alcohol history	-	-	1.804 (1.382–2.355)	<0.001
Uric acid (per 1 mg/dL)	-	-	5.337 (4.847–5.877)	<0.001
Hemoglobin (per 1 g/dL)	-	-	0.994 (0.950–1.041)	0.809
Total cholesterol (per 10 mg/dL)	-	-	0.996 (0.993–0.999)	0.015
LDL-cholesterol (per 1 mg/dL)	-	-	1.004 (1.001–1.008)	0.021
eGFR (per 1 mL/min/1.73 m^2^)	-	-	0.996 (0.994–0.999)	0.003
MetS	2.086 (1.849–2.354)	< 0.001	1.493 (1.312–1.700)	<0.001

Values are expressed as odds ratios and 95% confidence intervals (CIs). Abbreviations are the same as in Table 1. * Adjusted for age, sex, smoking and alcohol history, uric acid, hemoglobin, total cholesterol, LDL cholesterol, and eGFR.

**Table 3:** Associations between the sum of MetS components and new-onset hyperuricemia, determined using logistic regression analysis.

The Sum of MetS Components	Age and Sex Adjusted		Multivariable *	
	Odds Ratio (95% CI)	*p*	Odds Ratio (95% CI)	*p*
MetS number: 0	Reference		Reference	
MetS number: 1	1.853 (1.614–2.126)	<0.001	1.413(1.222–1.634)	<0.001
MetS number: 2	2.815 (2.438–3.249)	<0.001	1.918(1.647–2.233)	<0.001
MetS number: 3	3.262 (2.750–3.869)	<0.001	1.915(1.597–2.296)	<0.001
MetS number: 4	4.182 (3.326–5.258)	<0.001	2.428(1.901–3.100)	<0.001
MetS number: 5	5.357 (3.533–8.121)	<0.001	3.593(2.281–5.658)	<0.001

Values are expressed as odds ratios and 95% confidence intervals (CIs). Abbreviations are the same as in Table 1. * Adjusted for age, sex, smoking and alcohol history, uric acid, hemoglobin, total cholesterol, LDL cholesterol, and eGFR.

**Table 4:** Associations between MetS components and new-onset hyperuricemia, determined using multivariable logistic regression analysis.

Mets Components	Age and Sex Adjusted		Multivariable *	
	Odds Ratio (95% CI)	*p*	Odds Ratio (95% CI)	*p*
Abdominal obesity	1.353 (1.304–1.404)	<0.001	1.180 (1.134–1.229)	<0.001
Hypertriglyceridemia	1.427 (1.369–1.488)	<0.001	1.293 (1.233–1.355)	<0.001
Low HDL cholesterol	1.303 (1.254–1.354)	<0.001	1.185 (1.135–1.236)	<0.001
Hyperglycemia	1.126 (1.070–1.185)	<0.001	1.136 (1.075–1.201)	<0.001
High blood pressure	1.251 (1.202–1.303)	<0.001	1.167 (1.118–1.217)	<0.001

Values are expressed as odds ratios and 95% confidence intervals (CIs). Abbreviations are the same as in Table 1. * Adjusted for age, sex, smoking and alcohol history, uric acid, hemoglobin, total cholesterol, LDL cholesterol, and eGFR.

## Data Availability

The data underlying this study are from the Taiwan Biobank. Due to restrictions placed on the data by the Personal Information Protection Act of Taiwan, the minimal data set cannot be made publicly available. Data may be available upon request to interested researchers. Please send data requests to: Szu-Chia Chen, PhD, MD. Division of Nephrology, Department of Internal Medicine, Kaohsiung Medical University Hospital, Kaohsiung Medical University.
